# Livogrit, a herbal formulation of *Boerhavia diffusa*, *Phyllanthus niruri* and *Solanum nigrum* reverses the thioacetamide induced hepatocellular toxicity in zebrafish model

**DOI:** 10.1016/j.toxrep.2022.03.053

**Published:** 2022-04-29

**Authors:** Acharya Balkrishna, Savita Lochab, Anurag Varshney

**Affiliations:** a Drug Discovery and Development Division, Patanjali Research Institute, NH-58, Haridwar 249405, Uttarakhand, India; b Department of Allied and Applied Sciences, University of Patanjali, Patanjali Yog Peeth, Roorkee-Haridwar Road, Haridwar 249405, Uttarakhand, India; c Patanjali Yog Peeth (UK) Trust, 40 Lambhill Street, Kinning Park, Glasgow G41 1AU, UK; d Special Centre for Systems Medicine, Jawaharlal Nehru University, New Delhi, India

**Keywords:** Hepatotoxicity, Livogrit, Zebrafish, Thioacetamide, Herbal formulation, AST, Aspartate Transaminase, INR, International Normalized Ratio, HED, Human Equivalent Dose, ED, Effective Dose, MELD, Model for end-stage liver disease, TAA, Thioacetamide

## Abstract

Research studies in the past years have shown encouraging therapeutic potential of herbal medicines in liver ailments. Livogrit is a well characterized formulation prepared by mixing extracts of plants, *Boerhavia diffusa*, *Phyllanthus niruri* and *Solanum nigrum* in precise ratios. Our study demonstrates the curative role of Livogrit in thioacetamide (TAA) induced zebrafish model of hepatotoxicity. This is a systematic study, wherein we first screened Livogrit for an effective dose and treatment time-course. Once established, we conducted subsequent experiments to compare the hepatoprotective effects of Livogrit with a reference drug, prednisone. We evaluated a wide range of liver function variables including, albumin, AST, bilirubin, creatinine, platelet clotting factor, INR and sodium blood serum to assess the degree of liver dysfunctionality. Results from screening experiments suggested that Livogrit treatment for 14 days at an effective dose (ED3-142 μg/kg) significantly revamped the deviated serum biochemistry. The experiments comparing prednisone and Livogrit demonstrated that the treatment with the herbal formation was more effective against TAA-induced hepatotoxicity. Liver function parameters indicating hepatic dysfunctionality showed better recovery with Livogrit as compared to prednisone. Furthermore, we enumerated a scoring method for assessing degree of liver dysfunctionality based on the values of bilirubin, creatinine and INR. The herbal formulation in comparison to prednisone successfully restored the liver dysfunction index to low risk. The liver cytology showed a decline in the hepatocyte cell death that further corroborated the promising curative potential of Livogrit.

## Highlights

• Livogrit is a phytochemically characterized herbal formulation.
• Livogrit treatment reverts TAA-induced hepatotoxicity in zebrafish model.
• Livogrit progressively normalizes the deregulated liver function parameters.
• Liver dysfunction index minimized to low risk upon Livogrit treatment.
• Liver cytology corroborates the hepatoprotective potential of Livogrit.

## Graphical Abstract

**Figure:**
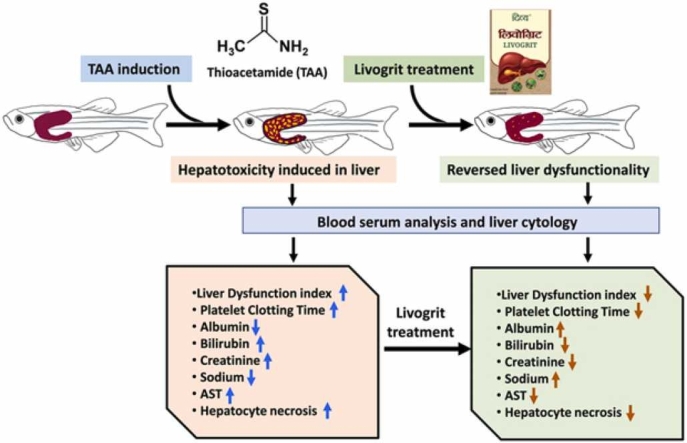
**Livogrit reverses the TAA induced hepatotoxicity.** The schematic model summarizing important finding from the current study. TAA induction induced severe hepatotoxicity in zebrafish model. Blood serum biochemistry and liver cytology were studied to evaluate the degree of liver dysfunctionality. Livogrit treatment post establishment of hepatotoxicity led to significant recovery of liver functionality.

## 1 Introduction

Liver is a functional hub to essential biological processes of digestion, nutrient metabolism, energy homeostasis, biotransformation of xenobiotics, maintaining blood volume and electrolyte balance [1]. A significant global rise in individuals claiming their lives to various liver disorders is a matter of serious concern [2]. Interconnected pathologies among different liver ailments ranging from liver cirrhosis, viral hepatitis, hepatocellular carcinoma to alcoholic or non-alcoholic fatty liver and drug-induced liver injury have led to complications in the treatment [3]. Sensitive diagnosis and better treatment strategies are needed to impede the increasing morbidity and mortality of chronic liver diseases every year.

Overwhelming usage of drugs and alcohol is a prominent factor predisposing a significant number of individuals towards hepatitis and liver cirrhosis. Moreover, hepatoxicity due to environmental toxins also increase the rate of progression of liver injury [4]. In the recent years, traditional and complementary medicine system have gained popularity in preventing and treating complex diseases of liver [5], [6]. A thorough scientific and clinical research is still needed to further substantiate the hepatoprotective activity of traditional medicines. In this study, we have explored the therapeutic potential of an Ayurvedic medicine, Livogrit (also known as Divya-Sarva-Kalp-Kwath). The formulation is prepared by mixing aqueous extracts derived from three plants, *Boerhavia diffusa L.* (Nyctaginaceae), *Phyllanthus niruri L.* (Euphorbiaceae), and *Solanum nigrum L*. (Solanaceae) in 2:1:1 ratio, respectively and have been earlier shown to effectively revert carbon tetrachloride (CCl_4_)-induced hepatocellular injuries in rats and HepG2 cells [7]. The study, indeed chemically characterized Livogrit using Liquid Chromatography-Mass Spectroscopy (LC-MS-QToF) and identified a substantial presence of gallic acid, citric acid monohydrate, gentisic acid, brevifolincarboxylic acid, catechin, caffeic acid, ellagic acid, corilagin, rutin, coccineone B, kaempferol, solasodine, apigenin, naringenin and quercetin in addition to 53 other metabolites [8]. Our subsequent study corroborated the previous one with mechanistic insights and showed that Livogrit reduces the intracellular triglycerides and extracellular glycerol levels to inhibit the development of fatty acid-induced steatosis in HepG2 liver cells [9]. In a recent study we have shown that Livogrit recovered HepG2 cells from alcoholic steatosis at biochemical and molecular levels by modulating the transcription levels of SREBP1c, FAS, PLIN2, TNF-α, NF-κB, and LC3A genes associated with lipogenesis, inflammation, and autophagy [7]. In our efforts to comprehensively address the hepatocurative potential of Livogrit, our current study has been conducted in zebrafish (*Danio rerio*) model of liver toxicity. The similarities towards mammals in terms of cellular mechanisms, enzyme activation, disease pathology, biochemistry during liver toxicity have established zebrafish as promising experimental model [10]. In the present study, we introduced hepatotoxicity in zebrafish with a fungicide, thioacetamide (TAA), an acknowledged mode of inducing experimental hepatotoxicity in various *in vivo* and *ex vivo* models [11], [12]. Although TAA administration is lethal to *in vivo* model organisms but researchers still get a wide window of time course to evaluate the mechanism of damage in hepatocytes. TAA-induced model organisms are generally exploited to address the recovery rate of a potential hepatoprotective compound.

In our study, we performed a time and dose dependent screening to determine an effective dose of Livogrit and ascertain a time frame that shows the recovery from hepatotoxicity. Transaminase enzymes, metabolites, proteins and electrolytes are reliable biochemical parameters to assess the functionality in liver metabolism [13]. Liver toxicants damage the hepatocytes that results in leaching of the enzymes, proteins or electrolytes into the systemic circulation that indicates hepatocyte injury. We performed biochemical analysis of total albumin, creatinine, aspartate transaminase (AST), bilirubin, platelet aggregation time and its international normalized ratio (INR) in the blood serum of TAA-induced zebrafish to determine the degree of liver abnormality. Once the hepatotoxicity was established, we screened zebrafish model for three effective doses, 6 μg/kg (ED1), 28 μg/kg (ED2) and 142 μg/kg (ED3) wherein timeline of treatment was 7 and 14 days. After 14 days, ED3 showed better recovery of liver function parameters compared to ED1 and ED2. Based on the altered bilirubin, albumin and creatinine levels, we then calculated liver dysfunction index indicating the degree of decompensated liver function. The elevated liver dysfunction index calculated in TAA-induced zebrafish reversed to the levels similar in normal control zebrafish. Imbalance in the sodium levels due to TAA was reverted back to normal in zebrafish treated with Livogrit. Furthermore, the hepatoprotective potential of Livogrit was compared to prednisone, a standard reference drug used to treat impaired liver function. Livogrit as compared to prednisone showed better reversal of the liver function index to low risk. Moreover, Livogrit significantly reduced the hepatocyte necrosis in liver of TAA-induced zebrafish. Our study demonstrates that the tri-herbal formulation, Livogrit effectively restores the aberrant biochemistry in blood serum due to hepatotoxicity.

## 2 Materials and methods

### 2.1 Chemicals and reagents

Livogrit was manufactured and procured from Divya Pharmacy, Haridwar, India. Thioacetamide, prednisone, Haematoxylin & Eosin were purchased from Sigma-Aldrich (Merck), Bangalore.

## 3 Animals

Adult Wild type *AB* strain zebrafish were sourced from Pentagrit in-house breeding facility. Zebrafish were randomly selected from any gender with comparable weight and age (1.2-1.5 years). Each study group had 24 zebrafish in polypropylene tank maintained at 27 ± 1 °C with 14 h:10 h (light: dark) circadian cycle. Zebrafish was fed 5 mg per gram body weight of commercial feed (TetraBit, Spectrum Brands Pet LLC, Blacksburg, VA, USA) everyday [14]. All the experimental procedures and protocols with respect to zebrafish were approved by the Institutional Animal Ethical Committee (IAEC approval number- 220/Go112019/IAEC) and were followed according to the guidelines of Committee for the Purpose of Control and Supervision of Experiments on Animals (CPCSEA), Government of India; and were in compliance with ICH harmonized principles for animal housing and handling.

**Fig. 1:**
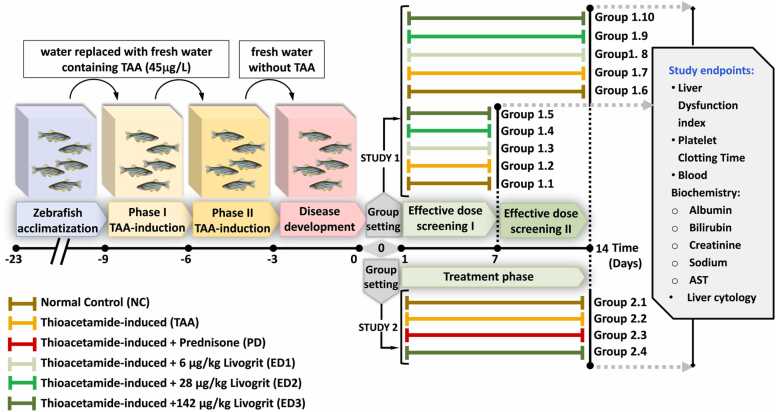
**Study design illustrating the experimental set ups.** Experimental set up indicating study 1, was performed to screen Livogrit for evaluating the effective dose and in TAA-induced zebrafish model. The time course of the treatment was continued for 7 days (effective dose screening I and II) and 14 days of group setting. Study 2 is a second experimental set up to assess the comparative therapeutic effects of Livogrit with another reference drug, prednisone post 14 days of treatment.

**Study design**: Our study includes two experimental set ups. The first experimental design was established to screen effective dose of Livogrit in thioacetamide treated zebrafish. Second experimental set up evaluated biochemical and cytological endpoints in thioacetamide-induced zebrafish model treated with therapeutic dose of Livogrit. Zebrafish were acclimatized for 14 days under standard laboratory conditions. Zebrafish were induced with thioacetamide in two continuous phases. Initial dose (-9th day) of 45 μg per litre was added to each experimental tank except the normal control group. After 2 days (-6th day), the tank water was again replaced with thioacetamide containing water at the concentration of 45 μg per litre and maintained as such for 3 more days (till -3rd day). Zebrafish were then transferred to regular water conditions without thioacetamide for 3 more days (till day 0). From day 1, zebrafish were fed with diet pellets incorporating 3 different doses of Livogrit (6 µg/kg, 28 µg/kg and 142 µg/kg). Study groups were screened for non-invasive biochemical markers and cytology in liver tissue for assessing damage at day 7 and 14. The normal control groups were maintained in regular housing conditions throughout. In the second experimental set up, zebrafish were induced with thioacetamide as described above and fed with diet composed of either therapeutic dose of Livogrit (142 µg/kg) or Prednisone (0.6 µg/kg) for 14 days. Prednisone is prescribed for the treatment of active liver disease [15]. Experimental design and group settings are illustrated in Table 1 and Fig. 1.

**Table 1:** Layout of the study groups, treatment and duration of treatment.

		Group No.	Treatment
**Study 1**	**7 Days**	1.1	Control
		1.2	TAA
		1.3	TAA + Livogrit (6 µg/kg)
		1.4	TAA + Livogrit (28 µg/kg)
		1.5	TAA + Livogrit (142 µg/kg)
	**14 Days**	1.6	Control
		1.7	TAA
		1.8	TAA + Livogrit (6 µg/kg)
		1.9	TAA + Livogrit (28 µg/kg)
		1.10	TAA + Livogrit (142 µg/kg)
**Study 2**	**14 Days**	2.1	Control
		2.2	TAA
		2.3	TAA + Prednisone (0.6 µg/kg)
		2.4	TAA + Livogrit (142 µg/kg)

## 4 Drug dosing

We optimized the drug administration for zebrafish at 1000 times less dose compared to the respective human dose [16]. Recommended human dose of Livogrit is 28.5 mg per kg body weight per day (2 g/day) was translated to 28 µg per kg body weight each day for zebrafish. To screen effective dose of treatment, zebrafish were treated with three effective doses ranging from ED1, ED2 and ED3, details of them are briefed in Table 2. One dilution below HED (0.2X) and one dilution above HED (5X) were preferred for determining pharmacological impact of Livogrit. HED for prednisone is 40 mg/day (0.6 mg/kg/day) that was translated into 0.6 µg/kg/day for zebrafish. All doses of Livogrit and prednisone were administered orally by mixing per day dose the food pellet. Fish were individually fed upon the modified food pellet in a separate feeding tank to monitor that entire per day dose is consumed by every fish in all groups. Fish in the control group were fed with unmodified food pellet.

**Table 2:** Human equivalent dosage of Livogrit and Prednisone in zebrafish.

Prednisone		Livogrit			
Treatment		Treatment Group	Treatment		Dose translated in μg/kg/day
Human DoseHED	40 mg/day 0.3 ng/day/fish(0.6 μg/kg/day)	- ED1 ED2 ED3	Human DoseHED (0.2X)HED (1X)HEX (5X)	2 g/day 3 ng/day/fish 14 ng/day/fish 71 ng/day/fish	6 μg/kg/day28 μg/kg/day142 μg/kg/day

HED: Human Equivalent Dose in zebrafish; ED: Effective Dose

## 5 Blood collection from zebrafish

Fish were euthanized through rapid cooling method discussed previously [17]. The blood was collected immediately by making incision in the region of dorsal aorta and inferior vena cava, just posterior to the dorsal fin near the caudal region. Smear were promptly prepared on the glass slides preloaded with 0.5% EDTA. The rest of the blood was collected and centrifuged to separate plasma for biochemical analysis.

## 6 Biochemical assays

We investigated albumin, aspartate transaminase (AST), bilirubin, creatinine and sodium levels in the diluted blood serum of zebrafish using biochemical kits procured from Randox Laboratories, Crumlin, United Kingdom. We adhered to the protocols accompanying the kits for analysis of all the biochemicals.

## 7 Platelet clotting time

Blood clotting time was calculated according to manufacturer’s protocol provided with the kit. Based on the platelet aggregation time obtained for each zebrafish, international normalized ratio (INR) was calculated.

## 8 Evaluation of liver dysfunction index

Various methods have been adapted to describe severity of liver decompensation. These methods that are used to score liver dysfunction consider subjective and preferably objective variables. Model for end-stage liver disease (MELD) scoring method is one of the widely accepted and commonly used prognostic methods to predict the survival of patients with liver complications [18]. However, zebrafish liver toxicity model lacks a standard method for scoring liver dysfunctionality. Therefore, we calculated liver dysfunction index in zebrafish by modifying model for end-stage liver disease (MELD) scoring method. The minimum limit in MELD score is fixed at 1 to avoid negative scores but in case of zebrafish we preferred not to alter the obtained values. For that normalization of datasets through log transformation was precluded in zebrafish model. In addition, we chose not to include the subjective elements in determining the score and only considered the objective parameters [18]. The purpose of liver dysfunction index was to obtained a reference and comparative score of liver dysfunctionality that considers more than one biochemical parameter. Liver dysfunction index utilizes the absolute biochemical readings of bilirubin, creatinine, INR in each zebrafish of all groups. Following formula was used for calculating liver dysfunction index:

Liver dysfunction index = (9.57* Creatinine) + (3.78* Bilirubin) + (11.2 INR)

## 9 Liver cytology

Fish were euthanized and dissected according to the guidelines of animal ethics committee. Liver was dissected out using sterilized surgical equipment from zebrafish from all study groups albeit sequentially and were placed in PBS for 1 min. The liver can be identified by its large size, lobed morphology, tannish colour, and extensive vascularization. Liver was then smeared on a glass slide and was allowed to air dry. Progressive staining with Haematoxylin followed by Eosin was used for better definition of nucleus and the cytoplasm. Slides were observed under bright field 40X magnification using 1×400 microscope (Labomed, Los Angeles, CA, USA). For quantification of number of dead hepatocytes in each slide, a total of 300 cells in three fields were analyzed for nuclear morphology in each zebrafish of all groups [19]. Percentage of dead cells with respect to normal control group was calculated and plotted such that each circle represents percentage of dead cells in individual zebrafish. One area per image have been zoomed into to show the normal versus dead cells as the slides prepared from the liver smear have non-specific background elements.

## 10 Statistical analysis

Statistical analysis was done using Prism 8 software from GraphPad. The means of two unmatched groups were compared with the assumption that the values followed a Gaussian distribution. Dunnett’s multiple comparison tests of groups, a one-way ANOVA at an alpha = 0.05 (95% confidence interval) was applied to all datasets except INR and Liver Dysfunction Index. The later, are considered as scores, therefore, Friedman non-parametric test was applied these datasets [20]. Data sets of each group are expressed as mean ± standard error of mean (SEM). p-values for the data sets were considered significant if p<0.05 (*p<0.05, **p<0.005, ***p<0.0005) and not significant (ns) if p>0.05

## 11 Results

### 11.1 Deregulated liver function parameters progressively normalize with Livogrit treatment

**Fig. 2:**
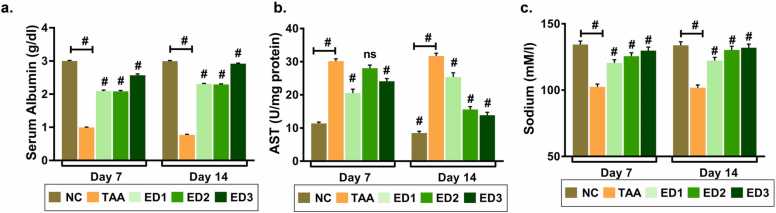
**Deregulated liver function parameters progressively normalize with Livogrit treatment.** Blood serum samples collected from study 1 were subjected to biochemical tests. Bar graph representing the (a) average serum albumin levels (g/dl); (b) AST levels (U/mg of protein); (c) sodium levels (mM) of each study group post 7 and 14 days of treatment. Error bars represent ±SEM; significance of data represented as, **#** p<0.0001 and not significant (ns) if p>0.05. The # representing over bars in group ED1, ED2 and ED3 was calculated with respected to TAA. NC: normal control group, TAA: TAA treated group, ED1: TAA+6 µg/kg Livogrit, ED2: TAA+28 µg/kg Livogrit, ED3: TAA+142 µg/kg Livogrit. Number of zebrafish per group, n=24.

Liver function parameters reflect the abnormalities associated with liver functionality. Advanced liver cirrhosis has been associated with not only reduced serum albumin but also with the quality of albumin [21]. Similarly, aberrant levels of prognostic enzyme, aspartate aminotransferase (AST) mark the presence of hepatocellular injury. We determined that average serum albumin levels significantly reduced to 1.0 g/dl and 0.8 g/dl from 3.0 g/dl post 7 and 14 days of TAA-induction, respectively (Fig. 2a). AST levels on the other hand elevated approximately 20 times in TAA- induced zebrafish in comparison to control zebrafish (Fig. 2b). A substantial decline in serum sodium levels because of TAA induction also indicates development of systemic imbalance of electrolytes (Fig. 2c). Although, TAA- induced zebrafish were treated with three different doses of livogrit but ED3 (142 µg/kg/day) dosage was most effective and showed better recovery in reduced albumin and sodium levels post 14 days of treatment. AST levels that were elevated significantly reduced in zebrafish that were treated with livogrit at ED3. Notably, progressive reversal of AST level was observed from 7 days to 14 days of livogrit treatment indicating livogrit is most effective at ED3 dose for 14 days.

## 12 Livogrit dose dependently minimize the liver dysfunction index to low risk

**Fig. 3:**
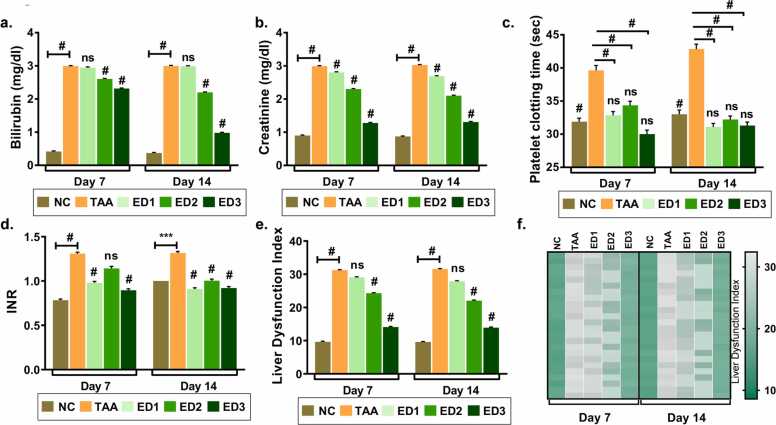
**Livogrit dose dependently minimize the liver dysfunction index to low risk.** Blood serum samples collected from study 1 were subjected to biochemical tests. Bar graph representing the (a) bilirubin levels (mg/dl); (b) creatinine levels (mg/dl of protein); (c) platelet clotting time (sec) of each study group post 7 and 14 days of treatment. (d) International normalized ratio was calculated as: platelet clotting time of treated group / platelet clotting time of control group) ^ISI^. (e) Liver dysfunction index calculated using bilirubin, creatinine and INR values of each group. (f) heat map depicting the liver dysfunction index. Each block of the heat map shows the liver dysfunction index in each zebrafish. The scale bar towards the right depicts the range of liver dysfunction index. Error bars represent ±SEM; significance of data represented as, **#** p<0.0001 and not significant (ns) if p>0.05. Significance in all graphs is calculated with respect to TAA unless indicated. In (c) the # representing over bars in group ED1, ED2 and ED3 was calculated with respected to TAA while ‘ns’ is calculated with respect to normal control group. NC: normal control group, TAA: TAA treated group, ED1: TAA+6 µg/kg Livogrit, ED2: TAA+28 µg/kg Livogrit, ED3: TAA+142 µg/kg Livogrit; number of zebrafish per group, n=24.

We derived liver dysfunction index as a score to assess the degree of abnormal functionality in zebrafish liver that indeed, is dependent on biochemical variables, bilirubin, creatinine and platelet clotting time. We assessed their levels after 7 and 14 days of livogrit treatment at ED1, ED2 and ED3 dosage of administration. Bilirubin and creatinine showed significant elevation upon TAA induction which subsequently reduced dose dependently with livogrit treatment (Fig. 3a-b). Although we observed that zebrafish were responsive to all doses of treatment but better reversal of bilirubin and creatinine levels were observed at ED3 dosage after 14 days of treatment. In line, we also determined the impact of livogrit treatment on platelet clotting time and INR in TAA-induced zebrafish (Fig. 3c-d). All the three doses, ED1, ED2 and ED3 of Livogrit were effective in maintaining the platelet clotting time and INR within 7 days of treatment. To arrive at a conclusion on single dose and time course of treatment, we derived liver dysfunction index that would take into account values of three variables, bilirubin, creatinine and INR. Liver dysfunction index indicate that Livogrit at ED3 was most effective in minimizing the biochemical abnormalities (Fig. 3e). ED3 indeed was effective after 7 days of treatment. ED1 and ED2 were more effective after 14 days of treatment. We further represented the liver dysfunction index in individual zebrafish of all groups. Heat map indicates a clear reversal of liver functionality from abnormal to normal after 7 and 14 days of Livogrit treatment at ED3 dosage (Fig. 3f).

## 13 Livogrit treatment at effective dose, ED3 significantly reduces cell death in hepatocytes

**Fig. 4:**
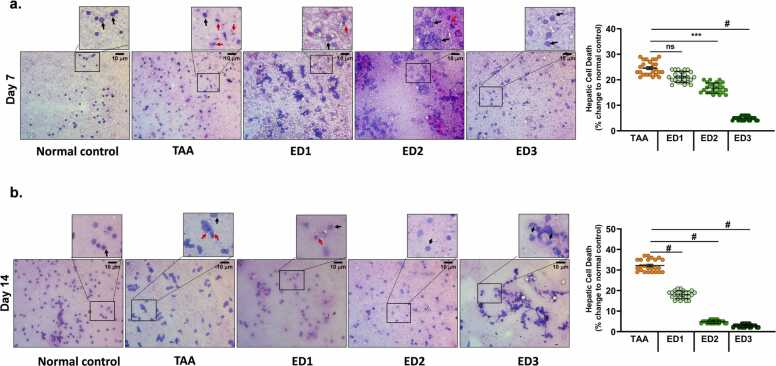
**Livogrit treatment at effective dose, ED3 significantly reduces cell death in hepatocytes.** Representative images of the smear cytology of liver showing well-defined hepatocytes in NC while study groups, TAA, ED1, ED2 and ED3 showing red arrow towards dead cells. Images of the smear cytology corresponds to the study group 1 wherein the groups in the (a) upper panel received livogrit treatment for 7 days and (b) lower panels were subjected to 14 days of Livogrit treatment. Each image has a box marked within, that is further zoomed in to clearly show the normal (black arrow) and dead hepatocytes (red arrow). For quantification of number of dead cells, a total of 300 cells from three different fields were analyzed for nuclear morphology in each zebrafish. Percentage of dead cells with respect to normal control group was calculated and plotted wherein each circle represents percentage of dead cells in individual zebrafish. Graphical representation depicts the percentage of dead cells that is normalized with respect to normal control group after (a) 7 and (b) 14 days of livogrit treatment, respectively. Error bars represent ±SEM; significance of data represented as, **#** p<0.0001. The significance over bars in group ED1, ED2 and ED3 was calculated with respected to TAA. NC: normal control group, TAA: TAA treated group, ED1: TAA+6 µg/kg Livogrit, ED2: TAA+28 µg/kg Livogrit, ED3: TAA+142 µg/kg Livogrit. number of zebrafish per group, n=24.

Smear cytology of liver shows well-defined hepatocytes and none of the dead or degenerative cells in zebrafish of the normal control group. Nearly 20 to 30% more dead cells were observed in TAA-induced zebrafish compared to control group (Fig. 4a-b). Cytology suggests that ED3 dose for 14 days is most efficient in restoring the healthy hepatocytes and reducing the toxicity effects of TAA. Dose dependent treatment showed significant reduction in the percentage of cell death which was more evident after 14 days of livogrit treatment (Fig. 4b).

## 14 Parameters indicating hepatic dysfunctionality resume normal levels upon Livogrit and reference drug treatment

**Fig. 5:**
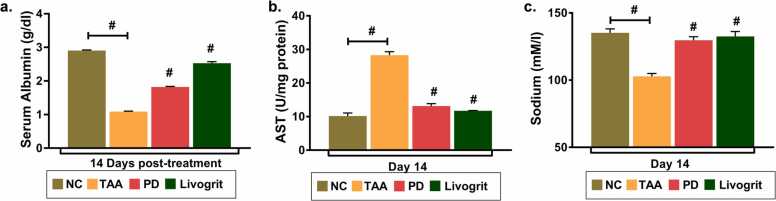
**Parameters indicating hepatic dysfunctionality resume normal levels upon Livogrit and reference drug treatment.** Blood serum samples collected from study 2 were subjected to biochemical tests. Bar graph representing the (a) average serum albumin levels (g/dl); (b) AST levels (U/mg of protein); (c) sodium levels (mM) of each study group post 14 days of treatment. Error bars represent ±SEM; significance of data represented as, **#** p<0.0001. The significance over bars in group ED1, ED2 and ED3 was calculated with respected to TAA. NC: normal control group, TAA: TAA treated group, PD: TAA+ 0.6 µg/kg prednisone, Livogrit: TAA+ 142 µg/kg Livogrit; number of zebrafish per group, n=24.

Serum albumin and AST levels are one of the important parameters of liver function test. Abnormal levels compared to normal zebrafish were observed upon TAA induction. Prednisone, is a standard drug used against chronic liver ailments. In our study we have used prednisone as a standard drug to compare the reversal effects of livogrit in TAA-induced zebrafish. Prednisone recovered the serum albumin level that was reduced to 0.8 g/dl upon TAA-induction to 1.8 g/dl. Livogrit on the other hand showed better recovery of albumin levels to 2.5 g/dl which was comparable to average albumin levels 2.9 g/dl in normal control group (Fig. 5a). AST levels that enhanced abruptly from 10 to 28 U/mg protein decline back to normal in groups treated with either prednisone or livogrit (Fig. 5b). Decline in the sodium levels of TAA-induced zebrafish group indicates electrolyte imbalance due to liver toxicity. Prednisone and livogrit recovered comparable sodium levels at 130 mmol/L and 135 mmol/L, respectively from 103 mmol/L in TAA-treated zebrafish group (Fig. 5c).

## 15 Livogrit, in comparison to reference drug effectively normalized the liver dysfunction index

**Fig. 6:**
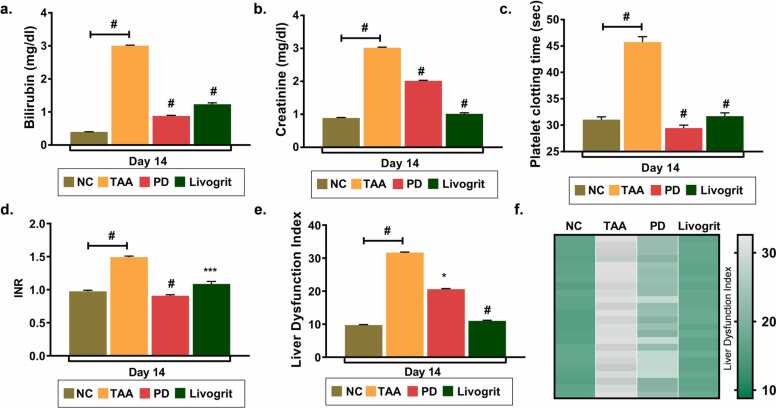
**Livogrit, in comparison to reference drug effectively normalized the liver dysfunction index.** Blood serum samples collected from study 2 were assessed for various biochemical parameters. Bar graph representing the (a) bilirubin levels (mg/dl); (b) creatinine levels (mg/dl of protein); (c) platelet clotting time (sec) of each study group post 14 days of treatment. (d) International normalized ratio was calculated as: platelet clotting time of treated group / platelet clotting time of control group) ^ISI^. (e) Liver dysfunction index calculated using bilirubin, creatinine and INR values of each group. (f) heat map depicting the liver dysfunction index. Each block of the heat map shows the liver dysfunction index in each zebrafish. The scale bar towards the right depicts the range of liver dysfunction index. Error bars represent ±SEM; significance of data represented as, **#** p<0.0001. The **#** representing over bars in group ED1, ED2 and ED3 was calculated with respected to TAA. NC: normal control group, TAA: TAA treated group, PD: TAA+ 0.6 µg/kg prednisone, Livogrit: TAA+ 142 µg/kg Livogrit; number of zebrafish per group, n=24.

Livogrit and prednisone treatment in TAA-induced zebrafish showed significant retrieval of bilirubin, platelet clotting time and INR. While Livogrit showed a better recovery of creatinine levels as compared to prednisone (Fig. 6a-d). To arrive at a coordinated conclusion, we determined the average liver dysfunction index and observed that the score is less than 10 in zebrafish belonging to normal control group. However, the score increased abruptly to 32 in zebrafish induced with TAA which indicates high degree of abnormality in liver function (Fig. 6e). Livogrit reversed the score back to 11 whereas prednisone could only be able to reduce it to 21. The result indicate that Livogrit regained the liver functionality as compared to prednisone treatment. Heat map indicates Livogrit ED3 effectively normalized the liver dysfunction index in all subjects of the groups in comparison to prednisone (Fig. 6f).

## 16 Livogrit shows decline in hepatocyte cell death in TAA-induced liver comparable to prednisone

**Fig. 7:**
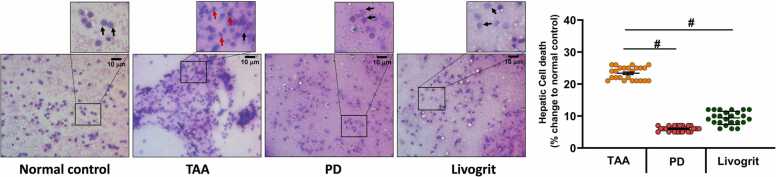
**Livogrit shows decline in hepatocyte cell death in TAA-induced liver comparable to prednisone.** Representative images of the smear cytology of liver from study group 2 indicating well-defined hepatocytes in NC while image from study group TAA showing red arrow indicating dead cells. Images of group treated with Livogrit and prednisone showing complete recovery of normal hepatocytes. Each image has a box marked within, that is further zoomed in to clearly show the normal (black arrow) and dead (red arrow) hepatocytes. Graph representing percentage of dead cells with respect to normal control group was calculated by quantitating a total of 300 cells in each zebrafish. Error bars represent ±SEM; significance of data represented as, **#** p<0.0001. The significance over bars in group PD and Livogrit was calculated with respected to TAA. NC: normal control group, TAA: TAA treated group, PD: TAA+ 0.6 µg/kg prednisone, Livogrit: TAA+ 142 µg/kg Livogrit; number of zebrafish per group, n=24.

We determined healthy hepatocytes in the liver cytology of control group zebrafish. TAA-induction zebrafish exhibited more than 20% higher cell death in hepatocytes than control indicating hepatotoxicity and progressive liver dysfunctionality. Study groups treated with prednisone or livogrit at therapeutic dose of ED3 showed significant decline in the cell death to 6% and 9%, respectively (Fig. 7).

## 17 Discussion

TAA is an established mode of inducing hepatotoxicity in experimental model organisms.

From chronic liver ailments, cirrhosis, hepatocellular carcinoma, hepatitis to drug induced liver injuries, alcohol induced liver damage, all ailments instigate severe hepatocellular damage. With an advancement in modern analytical techniques, extraction and identification of active molecules, real time toxicokinetics, high-throughput cell-based and cell free assays have led to remarkable surge in the scientific studies exploring traditional and complementary medicines [22]. Silymarin, Liv 52, Glycyrrhizin, and extracts from genus *Phyllanthus* are few exemplars to this [23]. Our group has been working extensively in this field and has previously shown that Divya-Sarva-Kalp-Kwath (also called as Livogrit) was effective against hepatocellular toxicities induced by carbon tetrachloride (CCl_4_) in rats [8]. We also dissected the mechanistic details and showed that SKK reduces the intracellular triglycerides and extracellular glycerol levels to inhibit the development of fatty acid-induced steatosis in HepG2 liver cells [9].

Our current study has investigated the hepatoprotective role of Livogrit in zebrafish model. Chronic hepatotoxicity can serve as a root cause of almost all sort of liver disorders. Drugs, medicines, exposure to environmental toxins, alcohol uptake are major reasons behind hepatotoxicity. Hence, to address the hepatoprotective activity of Livogrit we induced zebrafish with a well acknowledged hepatotoxin, TAA. We conducted time and dose dependent studies to determine the rate of recovery of liver dysfunctionality with Livogrit. We initiated the current study with an elaborative study design wherein zebrafish were treated with Livogrit after development of liver toxicity. Herbal medicines are known to have preventive role in disease development but our experimental design aim to study the therapeutic potentiality rather than prophylactic. We have previously tested safety assessments of Livogrit in zebrafish (data not shown), Wistar rats [8] and HepG2 cell lines [9]; therefore control groups with Livogrit treatment alone are not included in the experimental design (Fig. 1). Liver function tests are widely used to investigate even the asymptomatic liver dysfunctionalities. Consistent abnormalities in liver function tests are indicative of progressive liver disease. We assessed the hepatotoxicity mediated through TAA with liver function tests in the blood serum of zebrafish. Serum albumin levels in the blood serum have high prognostic value [24]. It is moreover a crucial parameter to assess the Child-Pugh-Turcotte score to ascertain the mortality risk in patients with liver cirrhosis [25].

A 2-fold reduction in TAA-induced zebrafish indicates severely impaired hepatocellular function. Amino transaminases are essential liver enzymes in amino acid metabolism and at the same time sensitive clinical parameters to evaluate liver abnormality. Nearly 20-times increase in AST level was observed due to TAA-induction in the blood serum that indicates substantial hepatocellular injury. AST has been considered as a sensitive clinical parameter to evaluate liver abnormality that leeches out in the blood serum during hepatocytic injury. However, the rate of elevation is an indicative of hepatic injury and not proportional to the degree of abnormality therefore, we also assessed other biochemical parameters (Fig. 2).

Maintaining ionic homeostasis is essential to regulate osmolality within the body. Abnormally low sodium level in the blood serum is a usual observation in patients with liver cirrhosis [26]. Indeed, the decreasing sodium levels are correlated linearly with increasing waitlist mortality prior to liver transplantation. This has led to the incorporation of sodium levels in MELD score, now called MELD-Na [27]. Considering sodium as an important parameter, we evaluated the levels. A significant reduction in the latter upon TAA-induction suggested an imbalanced osmolality in the plasma, possibly an outcome of hepatotoxicity. Livogrit on all the three effective doses, ED1, ED2 and ED3 was responsive in balancing back the abnormal levels of albumin, AST and sodium; ED3, however, appeared to be most effective with 14 days of treatment (Fig. 2).

Elevated creatinine and bilirubin in the serum of liver cirrhosis patients is a common observation. With a significant reduction in both the parameters upon TAA administration point out at establishment of a severe liver injury. Livogrit dose dependently and time dependently restored the levels to normal in TAA-induced zebrafish. In addition, Livogrit alleviated the platelet clotting time that enhanced with TAA induction (Fig. 3). A single blood serum parameter is never recommended to evaluate degree of hepatic injury. Rather a thorough estimation of additional variables is practiced to conclude the level of liver dysfunctionality. Model for end-stage liver disease (MELD) score, the MELD combined with serum sodium concentration (MELD-Na) score, the integrated MELD (iMELD) score, the Child-Pugh score and recently added, the albumin bilirubin (ALBI) score is commonly used prognostic methods to predict the survival of patients with Liver complications [28], [29]. All these scoring methods consider more than one parameter to establish the severity. We utilized bilirubin, creatinine and INR to calculate liver function index in zebrafish model. The purpose was to incorporate more than one parameter and then assess the therapeutic potential of Livogrit. Calculating the average liver dysfunction index and also in individual zebrafish showed that Livogrit dose dependently recovered the dysfunctionality index. Cytology of liver was also done to visualize the cell death in hepatocytes. Livogrit remarkably reduced the percentage of cell death indicating the progressive cytology of liver towards normal (Fig. 4). Furthermore, we compared the hepatoprotective activity of Livogrit at ED3 with a reference drug, prednisone. In comparison to prednisone, Livogrit showed better recovery albumin and AST levels, important parameters of liver dysfunctionality (Fig. 5). Prednisone, however showed better reversal in bilirubin but failed to restore creatinine to the normal level. Both, livogrit and prednisone were effective in recovering the aberrant serum variables but to determine the overall impact comprising of more than one parameter, we built a scoring method based on MELD score. Liver dysfunction index showed a better reversal in response to Livogrit suggesting Livogrit imposes a better hepatoprotective effect as compared to prednisone in TAA-induced zebrafish (Fig. 6). The data was further corroborated with cytology of liver suggesting the same (Fig. 7). It is conceivable that liver histology could have further shown the recovery of necrotic and degenerative cells upon Livogrit treatment but H&E staining in liver cytology could not classify the dead cells into necrotic or degenerative cells. However, the observed recovery in terms of total dead cells by Livogrit, collectively suggest that Livogrit can effectively reverse the TAA-induced hepatotoxicity (Graphical abstract).

The hepatoprotective effect of Livogrit can be attributed to the phytoconstituents present in *Boerhavia diffusa, Phyllanthus niruri,* and *Solanum nigrum.* Hepatoprotective efficacy of *Boerhavia diffusa* in combination with other plant extracts have already been shown to be effective in curing liver damage in rats induced with alcohol and CCl_4_ [30]. A scientific study showed that *Solanum nigrum* reduced the severity of fibrosis in different mice and rat induced with TAA and ethanol, respectively [31], [32]. *Phyllanthus niruri* have an established record of exerting hepatoprotective role in diverse *in vivo* models induced with different hepatotoxins [33]. All the three herbal constituents in Livogrit possess a range of phytometabolites namely, flavonoids, rotenoids, quercetin, kaempferol, alkaloids, anthocyanins, lignans, tannins and steroidal glycosides that account for the anti-inflammatory, anti-oxidant and hepatoprotective activity [8]. Although the need of hour is to develop highly specific and sensitive diagnosis techniques, but a better treatment strategy is also needed to control the global health burden of liver diseases. Our study is an effort to identify a safe hepatoprotective formula that can revamp the toxic effects in liver. We conclude that Livogrit is an effective tri-herbal formula against the potent hepatotoxin, TAA.

## Funding

This presented work has been conducted using internal research funds from a non-commercial and non-profit Patanjali Research Foundation Trust, Haridwar, India.

## CRediT authorship contribution statement

A.B. provided broad direction for the study, identified and prepared the test formulations, generated resources and gave final approval for the manuscript; S.L. curated. analysed the data, wrote the manuscript, A.V. conceptualized and supervised overall studies, generated resources, critically reviewed and finally approved the manuscript. All authors have read and agreed to the published version of the manuscript.

## Declaration of Competing Interest

The authors declare the following financial interests/personal relationships which may be considered as potential competing interests: The test article was sourced from Divya Pharmacy, Haridwar, Uttarakhand, India. In addition to Patanjali Research Foundation Trust and University of Patanjali, AB holds an honorary managerial position in Divya Pharmacy Haridwar, India. Besides, providing the test article, Divya Pharmacy was, not involved in any aspect of this study. Divya Pharmacy, Haridwar India, manufactures and sells many herbal medicinal products, including Livogrit. SL and AV are employed at Patanjali Research Institute which is governed by Patanjali Research Foundation Trust (PRFT), Haridwar, Uttarakhand, India, a not for-profit organization. In addition, AV is an adjunct professor in Department of Allied and Applied, Sciences, University of Patanjali, NH-58, Haridwar-249405, Uttarakhand, India; and in the Special Centre for Systems Medicine, Jawaharlal Nehru University, New Delhi-110067, India. All other authors declare no conflict of interest.
